# Creativity, Resilience and Resistance: Black Birthworkers’ Responses to the COVID-19 Pandemic

**DOI:** 10.3389/fsoc.2021.636029

**Published:** 2021-03-25

**Authors:** Julia Chinyere Oparah, Jennifer E. James, Destany Barnett, Linda Marie Jones, Daphina Melbourne, Sayida Peprah, Jessica A. Walker

**Affiliations:** ^1^ Provost and dean of the faculty, Race, Gender and Sexuality Studies Department, Mills College, Oakland, CA, United States; ^2^ Institute for Health and Aging, University of California, San Francisco, CA, United States; ^3^ School of Nursing, University of California, San Diego, CA, United States; ^4^ Black Women Birthing Justice Collective, Oakland, CA, United States; ^5^ California Preterm Birth Initiative, University of California, San Francisco, CA, United States

**Keywords:** COVID-19, maternal health, birth justice, Black women, doula care, coronavirus–COVID-19

## Abstract

This article documents the experiences of Black birthworkers supporting pregnant and birthing people and new mamas during the first six months of the COVID-19 pandemic. Building on the methodology and outcomes of Battling Over Birth–a Research Justice project by and for Black women about their experiences of pregnancy and childbirth–the authors utilized a “community-based sheltered-in-place research methodology” to collect the narratives of Black birthworkers, including doulas, certified nurse-midwives (CNMs), homebirth midwives, lactation consultants, community health workers and ob/gyns. The article examines the impact of restrictions put in place by hospitals and clinics, including inadequate or inconsistent care, mandatory testing, separation from newborns, and restrictions on attendance by birth support people, including doulas. Birthworkers shared the innovative approaches that they have devised to continue to offer care and the ways that they have expanded the care they offer to make sure the needs of Black birthing people and new parents are being met during this uncertain time. The article also explores the threats to health, safety, and financial security faced by Black birthworkers as a result of the pandemic, and the overt and subtle forms of racism they had to navigate. Finally, it documents the sources of strength that Black birthworkers have found to sustain them at the frontlines of a maternal health care system in crisis.

## Introduction

In March 2020, as the world grappled with the severity of the COVID-19 pandemic and daily life as we knew it changed dramatically, hospitals and medical clinics across the United States scrambled to put into place regulations and policies to protect birthing people, newborns, and staff. Across the country, non-medical birthworkers found themselves shut out of hospitals, where, for a time, some hospitals were not even allowing partners or spouses to be present for births. In this article, we explore how Black birthworkers responded to the dangers and stressors of working on the frontline of the maternal health-care system during the early days of the COVID-19 pandemic. The unfamiliar and unprecedented nature and impact of the novel coronavirus meant there was limited research about the impact of the pandemic on maternal health and health care. Initial research focused on pregnant women, revealing the heightened medical risks associated with COVID-19 infection for pregnant individuals in general, and the disproportionate impact of the COVID-19 virus on Black and Latinx pregnant women in particular (Ellington et al., 2020). These findings are consistent with research into racial disparities in maternal outcomes prior to the pandemic, which demonstrated the pervasive racialized inequities that contribute to disproportionate maternal mortality for Black pregnant persons (Oparah et al., 2018; Petersen et al., 2019; Tangel et al., 2019). However, no research to date has been published on the perspectives of Black birthworkers attending and supporting births during the pandemic. Given prior research demonstrating the positive impact of Black birthworker support in mitigating obstetric racism, this is a significant omission (Guerra-Reyes and Hamilton 2017; Oparah et al., 2018; Davis 2019). This article shares the findings of a community-based, Research Justice project into the experiences, perspectives and creative strategies of Black birthworkers during the COVID-19 pandemic. The article also describes the innovative virtual “sheltered-in-place” methodology and the Research Justice lens that facilitated data collection with a vulnerable and overworked population during a time of crisis.

This study builds on Battling Over Birth, a prior study carried out by Black Women Birthing Justice (BWBJ). BWBJ was founded after two Black mothers in Oakland, California had traumatic and coercive pregnancy and birthing experiences. The two new mothers, together with local birthworkers and advocates, formed BWBJ as a grassroots community organizing, educational and advocacy group with the vision “that every pregnant person has an empowering birth and postpartum experience, free of unnecessary medical interventions and forced separation from their child, one that honors their autonomy and maintains dignity” (BWBJ, 2020a). The group decided to find out whether and why other Black women were having similar negative experiences and to discover factors leading to positive pregnancy and birthing experience and outcomes.

Between 2011 and 2015, BWBJ conducted a participatory action research study into the perinatal experiences of 100 Black pregnant people in California. The study found a culture of fear and coercion, based on the disproportionate maternal and infant mortality facing Black communities and fueled by some medical professionals who used fear as a tool to discipline racialized birthing women, their partners and birth support individuals. For example, birthing people are often forcibly guided into unwanted interventions by the threat that failure to follow directives could make the birthing person complicit in “killing” her baby if there is a negative birth outcome. While these types of threats are used across racial groups, Black birthing people often have a heightened awareness of the potential for intervention by police or social services if their baby does not survive, or even simply for failing to comply with medical recommendations. In the context of this climate of coercion, Black pregnant people found themselves battling the very professionals whom they depended on during the vulnerable perinatal months. The study revealed the role of Black birthworkers, particularly doulas and midwives, in creating positive birthing experiences and outcomes for Black pregnant people. In keeping with the focus on action as a key part of a Research Justice approach, BWBJ launched the results of the study through a social media campaign using the hashtags #BattlingOverBirth and #LiberateBlackBirth, and employed the findings to raise awareness among Black communities, medical professionals, traditional birthworkers, media and legislators and to make recommendations for change.

BWBJ’s efforts are part of a national Birth Justice movement. Seeded in the wider Reproductive Justice movement, and reflective of Black women’s leadership, the Birth Justice Movement aims to “to dismantle inequalities of race, class, gender and sexuality that lead to negative birth experiences” and includes “the right to choose whether or not to carry a pregnancy, to choose when, where, how, and with whom to birth, including access to traditional and indigenous birth-workers, such as midwives and doulas, and the right to breastfeeding support” (BWBJ, 2020b). Birth Justice work is described as: “educating the community, and challenging abuses by medical personnel and overuse of medical interventions … (as well as) advocating for universal access to culturally appropriate, women-centered health care” (ibid).

Initially made up of grassroots organizations separated by geography and often working in isolation from one another, the Birth Justice movement gained national cohesion and visibility after the founding of the Black Mamas Matter Alliance in 2015 and the publication of Birthing Justice, an anthology of writings by scholars, birthworkers, activists and mamas that generated greater access to the stories and analyses underpinning the movement (Oparah and Bonaparte 2015). Black birthworkers have been at the forefront of this movement, working closely with pregnant and parenting people. As frontline workers who witness firsthand the “battle over birth” that often characterizes Black perinatal experiences, Black birthworkers aim to transform the conditions that lead to disproportionate Black maternal death and trauma. These efforts have led to significant gains, including the declaration of Black Maternal Health Week by Congress, the establishment of a Black Maternal Health Caucus and the development of the “Momnibus,” a slate of bills that together would expand and diversify the perinatal workforce, extend postpartum care, and invest in the social determinants of health and in community based organizations serving Black pregnant persons, et alia (Black Maternal Health Caucus, 2020). At the local level, birth justice advocacy has driven greater accountability and transparency by hospitals, funding for programs to provide doulas for low-income women and women of color, and greater awareness about the rights of pregnant and birthing persons.

In March 2020, as the COVID-19 pandemic ushered in a new era, many of the gains of the Birth Justice movement were summarily eliminated, as hospitals responded to the threat of the virus with swift and strict measures. In this article we seek to answer critical questions related to race, the COVID-19 pandemic, and maternal health care. How has the pandemic impacted a community already subject to racialized birth trauma and avoidable death? How are policies adopted to safeguard community health and reduce virus transmission impacting birthworkers’ ability to serve their clients? And how are Black birthworkers pushing back against rules and procedures that undermine their autonomy or negatively impact perinatal outcomes? We end our article with a call to action informed by the insights and advocacy of Black birthworkers and fueled by the urgency of the Black Lives Matter Movement.

## Methods

### “By Us Not For Us”: Research Justice During Covid-19

In March 2020, BWBJ began to hear stories about how the COVID-19 pandemic was impacting Black birthing people and birthworkers. As the stories streamed in—prenatal visits canceled, mothers being forced to give birth without any labor support, doulas being denied access to hospital maternity wards, laboring mothers waiting for COVID-19 test results in hospital lobbies—we decided to reconvene a community research team to document how Black maternal health care was being impacted by the pandemic. We called the research project Still Battling Over Birth to indicate a continuity between, before, and after the outbreak of the virus. For Black pregnant people, COVID-19 represents a crisis on top of a crisis: an already broken maternal health system attempting to deal with a life-threatening virus.

Given the troubled history of race and research, our research collective utilizes the “Research Justice” framework first articulated by the Oakland-based grassroots research organization Data Center in order to disrupt the inequitable racial dynamics of traditional social scientific and medical research (Assil et al., 2015; Oparah et al., 2015). Further articulated by Jolivétte (2015) in the edited collection Research Justice: Methodologies for Social Change, Research Justice builds on the principles of participatory action research (PAR), insisting on the interrelationships among theory, inquiry, reflection, and action, and re-imagining relationships between academic and community-based stakeholders in the research process.

While PAR has proven an effective tool for researchers wishing to directly engage impacted communities in the research process, it has also been critiqued for paying lip service to consulting communities while leaving traditional power dynamics and decision-making processes basically intact (Cooke and Kothari 2001). Research Justice seeks to overcome these limitations by advancing the concept of “community driven” research, whereby community members who have direct experiential knowledge and a personal stake in the problem being examined co-create the research agenda and collaboratively design the research process. Research Justice seeks to decolonize the research process by taking back power over research carried out in communities of color, eliminating hierarchies between academic and community researchers, and using research methods that honor the values and ethics of the community.

Yet, as we embarked on the project, we, the co-researchers, had to answer a number of methodological and ethical questions. How could we conduct meaningful community-based research online? How could we maintain the same ethic of care for participants as we did in person? Ultimately, we learned how to conduct what we call “community-based sheltered-in-place research” simply through the process of doing it.

While we also recruited and spoke with Black pregnant and birthing people for this project, this article focuses on the experiences of Black birthworkers, who are experiencing extraordinary pandemic-related stresses due to isolation, income loss, and potentially the illness or death of loved ones, while also seeking to meet the needs of a vulnerable population. In recognition of this context and of our ethical obligations to our research participants, we aimed to make participation in the research emotionally affirming and practically supportive. This study was reviewed and approved by the Mills College Committee for the Protection of Human Subjects in July 2020 and we began to reach out to birthworkers via email, social media, personal networks, and the BWBJ Black doula locator to invite them to share and listen during a 90 min virtual “sharing circle and strategy session.”

After birthworkers expressed interest in participating, they were sent an informed consent form via email. A member of our research collective reached out to review the consent form with them in detail. If participants agreed, they were offered the opportunity to participate in one of four sharing circles, held virtually over Zoom, based on their schedule and availability. Sharing circles were attended by 8-12 participants and were facilitated by two or three of the co-researchers (always including at least one birthworker). All co-researchers identify as Black women and were purposeful about creating a safe, healing space by opening the virtual circle with a spiritual, non-religious grounding, sharing group agreements, and providing caring and compassionate listening and support.

During the sharing circles, we placed a set of guiding questions in the Zoom “chat” (See Appendix A), and asked each participant to share her experience during the pandemic and/or the story of a birthing person she supported during the pandemic, with those questions in mind. Other participants were then invited to ask questions, suggest resources, or provide similar or contradictory experiences. There was also a time for group discussion, led by the interests and needs of the participants. In total, 38 birthworkers, including doulas, midwives, community health workers, and ob/gyns, participated in these sharing circles (see Table 1). After each circle, participating birthworkers were mailed a *Still Battling Over Birth* tote bag, designed by a local Black artist, and given three months free participation in BWBJ’s Birthworker Forum, a space for mutual support and information exchange.

**TABLE 1:** Sharing circle participants.

Characteristics	N	%
Race		
Black	38	100
Location		
Alabama	2	5.3
California	19	50
Delaware	2	5
Florida	1	2.6
Georgia	1	2.6
Maryland	1	2.6
New York	5	13.2
North Carolina	2	5.3
Ontario, Canada	1	2.6
Pennsylvania	2	5.3
Texas	1	2.6
Washington	1	2.6
Profession^a^		
Community Health Worker	7	18.4
Doula	23	60.5
Lactation Consultant	2	5.3
Midwife	3	7.9
Ob/gyn	3	7.9

^a^ Many birthworkers work in several different capacities (i.e., doula and lactation consultant). We have listed the profession the birthworker indicated is her primary role.

Sharing circles were recorded via Zoom and, first, automatically transcribed via Sonix.ai, and subsequently reviewed and corrected for accuracy by a member of our research collective. All members of our research collective read each sharing circle transcript. We met together to discuss themes that emerged to us based on the initial reading. These themes became our initial set of codes. Transcripts were analyzed using DeDoose qualitative analysis software. Three members of our team participated in coding. For each transcript, one member of TEAM would apply the pre-determined set of codes and another member would review. Any discrepancy in coding or added codes were discussed among the coders. After coding and review were complete, reports were generated of all quotes that fell under each code. These reports were reviewed by two to three members of the research collective (with at least one academic researcher trained in qualitative analysis reviewing each set of codes and quotes) for further refinement of themes. We met together to discuss this newly generated set of themes and continued to refine and analyze until we reached consensus on the most prominent themes in the data.

In the following sections, we explore the obstacles that birthworkers face as they strive to ensure Black pregnant and birthing people’s wellness and autonomy, how they are seeking to push back against COVID-19-related policies and procedures that undermine the effectiveness of their work or that they believe put their clients in danger, and how racism and racialized constructions of disease and risk are shaping their clients and their own experiences during the pandemic. We also explore the challenges to the birthworkers health and safety, emotional and mental wellness, and financial security, and document the innovative approaches and strategies they have developed in order to survive and thrive.

## Findings

### Uncertainty, Racialized Fear and Restrictions: The Impact of Pandemic-Related Policies and Procedures

*Just as a baseline, the medical model wasn't meeting the needs of Black women. I mean, we already know that, right? Like before COVID… So, it went from bad to worse, essentially.* (Ebony, midwife, California)

#### “So Many Mixed Messages”: Shifting Policies and Communication Gaps

As the realities of the pandemic became clear and, as also shown in other articles in this Special Issue, hospitals instituted new policies in an attempt to mitigate the spread of the disease. These policies were in constant flux, evolving alongside knowledge of COVID-19 and ebbing and flowing as cases rose and fell in different regions of the country. For many birthworkers, it seemed that “a lot of the policies in hospitals were kind of changing every day” and birthworkers spoke of the “anxieties that come with uncertainty and not knowing what space you're going to have to adapt to” (Imani, doula, Nevada).

Birthworkers noted that the needs of Black pregnant people and new mamas became casualties to a system struggling to pivot quickly to a predominantly online modality. In this context, continuity of care was disrupted, leaving pregnant people and new mamas without clear information on how to receive support:

[A]t least four or five people reached out to me not knowing who to contact about questions or just needing support getting visits. And that shouldn't have happened (Aliyah, ob/gyn, California)

In some cases, this lack of care resulted in neglect of medical conditions that should have received attention. One Birthworker spoke about a client who was “bleeding for no reason”:

So, she called me because she wanted me to go to the emergency room with her because they weren't listening to her. But … I couldn't go in with her to help advocate. So, I literally had to grab people and managers outside of the emergency room to... pretty much almost protest to get her to be heard. (Yolanda, community advocate, California)

Nia, a doula from Alabama, expressed concern for the safety of a client with a dangerous medical condition who was sent home postpartum with no immediate follow up visits; Nia said, “the problem that I'm having with this is that she's not being checked for the blood clots until six weeks after she has the baby.” Given the difficulties in accessing care or receiving clear and consistent information, Birthworkers became a critical source of support for pregnant people seeking knowledge about shifting hospital practices, support in accessing health care, and strategies for navigating hospital birth during the pandemic.

#### “I Couldn’t Really Support Her”: Impact of the “Doula Ban”

When the pandemic first started, nearly all hospitals initially banned the presence of any support person, then later, due in part to community protest, began to permit a maximum of one support person to be present during birth (Davis-Floyd et al., 2020). Since most women chose their partners over their doulas, this precluded doulas from attending most hospital births in person and has been incredibly disruptive to their practices and their ability to support birthing people. Since that time, some states have passed executive orders deeming doulas essential personnel who may attend births alongside a partner; however, the majority of states at the time of writing (January 2021) continue to allow hospitals to institute a *de facto* “doula ban.”

As a result, doulas have had to navigate an entirely new landscape of policies and regulations. Birthworkers reported that video calls during birth were perceived as a “liability” by medical staff and that the birthing person would have to advocate for the right to stay in touch with their doula during labor and delivery. Where doulas are allowed to physically attend births, restrictions on in-and-out privileges and the need to take turns with a partner result in unsustainable work hours and time spent in the lobby rather than the birthing room:

I think most families need both partners in the room, because he was the one that gave her the emotional support, that love that she needed, and I was there to help her do what she needed to do. But without both of us there, it made it very hard. (Janet, doula, Delaware)

Many birthworkers are uncomfortable with the forced shift to virtual support. Physical touch is seen as a vital component of traditional birthwork in the Black community:

[A]s the original Black auntie and the granddaughter of a midwife... touch is very important to me. So not being able to, like, reach out and touch and hug my clients, especially when you're in those formal spaces, has been very stressful. (Mariah, CNM, Delaware)

Even for those who were comfortable with offering care and support virtually, the logistics of engaging in new ways were often difficult to navigate. Teaching classes and demonstrating techniques over the computer required rethinking strategies. One birthworker spoke of attempting to offer a lactation consult over the phone to the parent of a baby in the NICU:

I asked her if we could FaceTime, but she had explained that, well, think about it. “If I’m in the NICU, I have the baby in one hand, I have the phone in the other hand.” It’s really hard to do a lactation consult with no extra help in the NICU. (Deja, lactation consultant, California)

#### “I Want My Baby in the Room”: Separation From Newborns

Several birthworkers noticed a disturbing pattern: hospitals were separating birthing people from their babies immediately after birth as a precaution, even when the new mama had not tested positive for COVID-19. As one doula, Imani, told us: “The thing that I’m most adamant about right now is the separation that I was noticing was happening. Doctor’s … haven’t really been encouraging skin-to-skin or any kangaroo care.” Noting that the American Academy of Pediatrics had recently revised their guidelines around newborn separation, Sienna, an ob/gyn, encouraged pregnant people to use this knowledge as an advocacy tool: “So even if the hospital policy hasn't changed, they can say, actually, your organization says that this is OK. And so, I want my baby in the room with me.”

This fear of separation plays a role in birthing people’s decision to consent to COVID-19 testing when they enter the hospital, as in most hospitals, a positive test would mean separation from the baby. Safiya, a community engagement coordinator from San Francisco, spoke of making this clear to her clients, saying, “yes, you have a choice not to be tested, but nobody tells you that if your baby ends up in the NICU (and you test positive), that you won't be able to be with your baby.”

#### “These Folks are Scared for Their Lives”: The Impact of COVID-19 Testing

A key tool that maternity wards deployed for pandemic mitigation was testing everyone who enters the facility for COVID-19. Yet both the process of testing and the potential results have implications for the birthing person and the birthworker. One birthworker noted that if her clients test positive, they are not allowed to have any support people attend the birth. She questioned whether this actually increased the safety of patients, providers or staff. Others echoed this concern and noted that if the birthing person tested positive, they could be separated from the baby after birth. Many birthworkers spoke of making sure their clients knew their rights and that “no one could test them against their will, because otherwise that was the pathway to seeing families separated” (Mariah, CNM, Delaware).

An unintended consequence of COVID-19 testing that frustrated several birthworkers in our study was the observation that hospital staff would not pay attention to the birthing person or her needs before they tested negative. Patricia, a doula from Philadelphia, said:

Before that COVID-19 test was clear, it was like, “We don't care that you haven't maybe felt the baby kick …” Everyone was just so concerned about whether she was positive and then that was going to determine the next level of care. So, you have people sitting in triage likely by themselves waiting for a COVID-19 test to return. And meanwhile, anything could be happening to them and that's not a concern.

For some, this extended beyond the time of the COVID-19 test. Tiara, a doula from Georgia, told the story of her own family’s experience with an ER visit where even after they tested negative they still felt that they were treated as carriers of the disease:

And it was just like even though we all tested negative, it still wasn't trusted that we were actually negative. And so, it's just like even if you're doing everything that you're supposed to do and you're not having the exact symptoms of COVID, there's the underlying belief that, you know, you're still a carrier and you could still be (infecting) others.

Many birthworkers understood the fear and risk at play for hospital workers. Yet, many perceived a particularly racialized fear of Black women. Black people were assumed to be carriers of the coronavirus, based on higher rates of infection in Black communities. Patricia, a Philadelphia doula, noted:

The media is painting us as being the carriers. Everyone in the hood is carrying it. We pass it on to each other. So, if Shaniqua come in to deliver, she got it, so y'all better step back. And Shaniqua's left to die in triage if she's complaining that something's going on, you aren't listening because you're waiting on her COVID-19 test to come back.

This racialized fear of diseased Black bodies has a long history within medicine and epidemiology. For example, Black people were considered to be tuberculosis carriers in the early 20th century, and fears of “the help” potentially infecting white children drove public health responses (Connolly and Gibson 2011). Yet, the idea that Black people are a threat to public health masks the real danger: that, due to structural racism, Black people are more likely to be exposed to the virus, and are at greater risk of serious consequences if they do contract COVID-19. As Dorothy Roberts asserted in an African American Policy Forum webinar: “It’s not race that is a risk factor for COVID-19, it’s racism African American Policy Forum, 2020.”

### “Picking Up the Slack”: Creative Strategies and Adaptations

Doulas have come up with creative strategies to work around the new barriers and regulations. Participants offered more prenatal visits, extended visits beyond six weeks postpartum, and maintained closer contact via phone, texting and video calls. Hospital restrictions have changed the nature of the work doulas do with their clients. Many birthworkers shifted from direct advocacy to encouraging birthing people and their partners to be their own advocates, even without the birthworker being present. Birthworkers have also found ways to encourage their clients to take charge of their own virtual prenatal care. One doula, Sahdiah, located in New York, discussed her clients:

I find that they're having a hard time transitioning to telehealth and so a lot of them don't know that they can get a blood pressure machine through their insurance. They don't know that they can get a scale so navigating that … ‘Cause you know, taking your blood pressure and doing your weight is self-advocacy.

Birthworkers have also taken on new tasks not usually in their scope of work, such as helping with grocery shopping, purchasing diapers, and providing personal protective equipment. This represents both a return to traditional modes of Black birthwork, as Delaware based doula Mariah described it “the way my granny used to,” and an innovation in the context of the dominant medical model:

[E]verybody's trying to figure out telemedicine and for our clients that don't have data plans, who rely on landline phones, who don't have Internet, who don't have computers, or if they do, they have bandwidth that's dedicated to their children just being in school. The idea that somebody would pick up a bag and bring a scale and a blood pressure cuff and a Doppler and come to your home is novel.

For Black birthworkers, this holistic carework is a form of resistance to racialized medical neglect. Black birthworkers have taken the crisis of COVID-19 as an opportunity to find new ways to form community, support new parents and share Black wisdom and healing traditions.

### “Who Would Take My Place?”: Surviving and Thriving During the Pandemic

As Black birthworkers seek to safeguard the emotional and physical safety and wellbeing of Black mamas and infants, they themselves face significant threats to safety and wellness. Black people have a higher risk of infection and are more likely to die from the virus once infected. This is not because Black people are biologically more susceptible to the virus, but “because we are more exposed and less protected” due to overrepresentation in essential jobs, jails and homeless encampments, inadequate healthcare and higher rates of chronic conditions related to health inequities (Ford et al., 2020; Wallis 2020). Black and Latinx healthcare workers run even greater risks (Jewett 2020). A recent study found that healthcare workers were three times more likely to report a positive COVID-19 test than the general public, and healthcare workers of color were twice as likely as their white peers to test positive (Nguyen et al., 2020). Black and Latinx healthcare workers are more likely to report using inadequate or reused protective gear and to care for patients with suspected or confirmed cases of the virus. They are also more likely to serve low income communities of color, which are particularly hard hit by the virus. For Black doulas, their risk may be magnified by having to work more than one job in order to make ends meet. For example, Jayla, a reproductive consultant in California, found herself exposed to the virus in her job as a transit worker:

When the pandemic hit, I was pregnant and considered an essential worker. I was not protected at work whatsoever... I had several passengers get on that tested positive for COVID. And when I voiced my concerns to the company as well as my health care provider, there was no care or concern about that, which I found, you know, shocking and to be honest, it was very infuriating also.

Mariah, a nurse-midwife serving Black families in Delaware, acknowledged the risk that she faces as an older Black woman in continuing to serve clients during the pandemic:

[B]ecause I am the only Black provider in the group space where I practice, I essentially live in complete isolation when I'm not with my clients because I know that if I get COVID-19 AND get sick and even if I'm just gone for two weeks, who will care for them? And if I'm one of the unfortunate Black health care providers who doesn't survive COVID-19, who would take my place?

Rather than worrying about her own welfare, Mariah expressed greater concern that her clients would not get their needs met should she, as the only Black midwife in her group, succumb to the virus. This spirit of service and self-sacrifice, while motivating birthworkers to continue to support a vulnerable population during the COVID-19 crisis, can also normalize unhealthy levels of stress, isolation and risk among these essential workers.

Racial isolation was another factor impacting the birthworkers in this study. The lack of interaction with other Black doulas, midwives and peers, was mentioned by several participants:

I'm the only Black midwife at the hospital...I do great work and I love this work. And I feel like I'm tied to my ancestors through it. But I also recognize that it is, like, physically and emotionally draining at times. And that being the “only” as many of us are, we don't always have someone to share it with. (Mariah, CNM, Delaware)

Several participants shared that the burden of navigating or speaking out against racism, and the climate of racial tension associated with reactions to the Movement for Black Lives, added to the stressors of the current moment.

#### “I Needed to Survive”: The Financial Impact of the Pandemic

When Black healthcare employees are faced with an unsafe or racially “toxic” environment, they face the choice of leaving and potentially facing financial insecurity, or staying in an environment that is detrimental to their physical and/or emotional wellbeing. Tamika, a lactation consultant who was pregnant during the pandemic, shared this dilemma:

I made a decision to take leave early, although I knew it would impact my family and my finances. But I needed to protect myself. I needed to survive. And I did not feel safe … it felt like I would for sure go into preterm labor if I had stayed in an environment that wasn't healthy or safe for me.

Unlike Tamika, most of the birthworkers who participated made the decision to continue practicing during the pandemic. Black doulas, midwives, and other birthworkers who operate independently may be faced with particularly acute financial challenges, as the pandemic causes furloughs and unemployment and simultaneously isolates women from spaces where they might otherwise have learned about independent maternal health professionals such as community midwives and doulas:

I have five clients right now, and four of the five are all people that I'm gifting support to. They're not paying. (Asha, doula, Washington)

[E]specially with the rise of unemployment... A lot of people aren't getting paid, so the ability for families and mothers to be able to compensate me for the services didn't really necessarily become my main goal. It was more so being able to assist in such a horrific time. (Imani, doula, Nevada)

The financial strain has been the most difficult on those birthworkers who work independently, with many noting a drop in income from either birthwork or other employment (many birthworkers also hold employment in other fields). Birthworkers working for clinics, hospitals or non-profits had a different experience and felt more stable through the first several months of the pandemic:

We haven't been hit that much financially because if the non-profits still have their grants in place or are getting reimbursements from a few state insurance programs, we're still able to take on as many families that qualify and can't truly afford [it]. (Patricia, doula, Philadelphia)

Several participants noted a surge in demand for home birth and home-based services, resulting in an increase in income for a small proportion of respondents. However, not all pregnant women who wished to birth at home could afford the full care of a midwife. In addition, many homebirth midwives do not have access to insurance and thus cannot bill for their services. In response to their clients’ financial challenges, several respondents participated in local grassroots fundraisers to pay for midwifery care for Black women who wished to give birth at home as a result of the pandemic.

#### “To Fight the Spiritual Battle”: Birthworkers’ Sources of Strength

Faced with significant financial insecurity, threats to their own health and safety, professional conflicts, racial and social isolation, and an intense sense of responsibility to serve and support Black pregnant people during the crisis, Black birthworkers are at risk of burnout or mental health challenges. Participants were conscious of the need to identify and draw on sources of strength to sustain them in their work. Nia, a doula from Alabama, found inner power by looking to a spiritual source:

[G]oing into yourself and really reaching down for that spiritual place for yourself in this, because it’s a lot … And there are so many negative forces around us right now in terms of COVID-19 that we don’t really understand or are able to see. And so, we have to go in knowing that we do have to fight the spiritual battle, because a lot of these doctors don’t have our best interest, of course.

When Black women turn to this metaphysical source, they are drawing on deep cultural wisdom that has sustained African American women’s resistance against insurmountable odds from slavery through the civil rights era and beyond. This cultural wisdom is passed down through oral and written testimonies and documentaries about the Black “granny” midwives (now called “Grand Midwives”) of the US South, who attended births for a century or more but were phased out of practice during the 1950s and 1960s by white doctors and health officials. Granny midwifery was considered a spiritual calling, and those called to serve could rely on a source of guidance and strength beyond their limited human resources in the context of overwhelming barriers to healthy Black births at a time when Black women were not admitted to hospitals (Smith and Linda, 1996; Susie, 2009; Turner, 2015). As we noted above, many Black birthworkers may be the only or one of only a few Black people working in maternal health in their communities. Connecting to this lineage of Black birthwork affords contemporary Birthworkers strength to carry on in the face of significant obstacles and isolation. Coming together with other Black birthworkers is another way in which participants in our study connected to this collective cultural wisdom; several participants responded to the pressure of their work by taking steps to reduce racial isolation—seeking out other Black birthworkers or participating in a Black Birthworker Forum:

It's a beautiful space and I really just encourage everyone, especially those of you who are feeling like you're the only [one], to be a part of it. Because you're not alone. And when you know you're not alone, it makes Monday or whatever day of the week that you think of when you're walking into a space that sometimes is unwelcoming, a different energy, because you know that these women are caring and these people are carrying you as you go. (Mariah, CNM, Delaware)

## Discussion

Our virtual sharing circles with Black birthworkers were not only an opportunity to share and compare experiences, but also a space to strategize for the future. Birthworkers provide care on the front lines of the pandemic. They hold the joy, the pain and the fears of their clients and offer a critical view on what is needed to support Black birthing people in a time of global crisis.

Birthworkers described to us the restrictions put in place by hospitals and clinics, including inadequate or inconsistent care, mandatory testing, separation from newborns, not allowing more than one support person during birth, and, in some cases, not even allowing doulas to support their clients virtually. Birthworkers have continued to provide care despite the restrictive policies and, in fact, have offered the vital service of helping their clients to navigate new policies and procedures as they emerge. They have found innovative ways to offer care and have expanded the care they offer to make sure the needs of Black birthing people and new parents are being met during this uncertain time. Moreover, they have done so in the face of overt and subtle forms of racism, through their own pregnancies and health challenges and, at times, without financial compensation. Black birthworkers saw the needs in their communities and rose to the challenges of fulfilling those needs in innovative ways that are grounded in the traditions of birthwork as a calling in the Black community.

Yet there is more work to be done. We have heard from birthworkers about what is broken and what, from their perspective on the frontlines, needs to change. Medical providers must ensure quality prenatal care and transparency and continuity of care throughout the perinatal period. At a time when perinatal visits are limited and virtual, and when postpartum support from other community networks, such as friends and family, may be inaccessible, it is more vital than ever that doulas, midwives, lactation consultants, and other birthworkers be compensated for the critical work they are doing, and that this care is extended beyond the standard 6–8 weeks postpartum period. By providing additional care, both during and after pregnancy, in addition to essential services like bringing groceries, diapers, and personal protective equipment, they are filling a gap that is always present, but wider than ever during the pandemic.

Hospitals must adopt policies allowing doulas to support birthing people in person. Each birthing person should have the right to be supported by whomever they choose and hospitals should facilitate safety protocols to allow this. Birth should center the comfort and safety of the birthing person over risk management concerns. This includes ensuring that birthing people of any color are not separated from their babies after birth. Black birthing people need access to the latest evidence and crucial resources to make pregnancy, birth, and postpartum successful. With mixed messages and shifting policies during COVID-19, many new parents don’t know where to turn or whom to trust.

Black birthworkers are providing care and serving their communities, not only in the context of a global pandemic, but also under the shadow of growing racial tension and increased focus on anti-Black state violence. All are aware of the disproportionate maternal and infant mortality rates for Black women and babies, and nearly all spoke of themselves and/or their clients experiencing racism in the course of giving or receiving care.

Restrictions and regulations in the time of COVID-19 have allowed for a resurgence of the racist and sexist policies that medicalized birth and pushed Black birthworkers to the margins. Black women’s bodies have continued to be seen as risky, both for pregnancy complications and for COVID, leading to a lack of care and touch that continues to put Black birthing people in danger. As one birthworker shared with us, “We can't just look at this as ‘during the pandemic’ type work. This is a battle in a war we must win.” While pandemic policies and regulations change, the core of the fight has stayed the same. The creative strategies and innovations proposed by Black birthworkers during the COVID-19 pandemic can be repurposed to transform birth during and beyond the pandemic. Black birthworkers are still battling over birth. Their resistance and resilience give us hope for the liberation of Black birth and for birth justice for all.

## Data Availability

Requests to access the datasets should be directed to Jennifer.James@ucsf.edu.

## Appendix A

### Sharing Circle Guiding Questions

1. What has it been like to support Black women in their pregnancy, birth and postpartum during COVID-19?
2. How is the prenatal, birth or postpartum support you provide impacted by safety procedures related to the COVID-19 pandemic?
3. What innovative strategies have you developed to care for your clients?
4. What ways have you seen Black mamas advocate for themselves during the pandemic? How has COVID-19 shifted birth choices for some mamas?
5. How are you resisting any COVID related policies or practices that negatively impact Black mamas and birthing persons? What structural or policy changes do you think are needed to support Black women during the pandemic?
6. What sources of community are available to Black pregnant and new mamas during shelter in place? How can we foster a sense of community?
