# Corrigendum: Creativity, Resilience and Resistance: Black Birthworkers’ Responses to the COVID-19 Pandemic

**DOI:** 10.3389/fsoc.2021.695303

**Published:** 2021-05-13

**Authors:** Julia Chinyere Oparah, Jennifer E. James, Destany Barnett, Linda Marie Jones, Daphina Melbourne, Sayida Peprah, Jessica A. Walker

**Affiliations:** ^1^ Provost and dean of the faculty, Race, Gender and Sexuality Studies Department, Mills College, Oakland, CA, United States; ^2^ Institute for Health and Aging, University of California, San Francisco, CA, United States; ^3^ School of Nursing, University of California, San Diego, CA, United States; ^4^ Black Women Birthing Justice Collective, Oakland, CA, United States; ^5^ California Preterm Birth Initiative, University of California, San Francisco, CA, United States

**Keywords:** COVID-19, maternal health, birth justice, Black women, doula care, coronavirus–COVID-19

## 

There is an error in the Authors Contributions statement. The correct Author Contributions should be stated as follows: “The article is the result of a community based research justice project involving a collaborative research team. All listed authors are members of the research team and participated in research design, participant recruitment, data collection and analysis. The article was co-written by JO and JJ. JO and JJ contributed equally to the manuscript.”

In the published article, there was an error regarding the affiliations for Linda Marie Jones, Sayida Prepah, and Jessica Walker. Instead of being affiliated with the University of California, all should have all been listed as affiliated with Black Women Birthing Justice. 

The authors apologize for this error and state that this does not change the scientific conclusions of the article in any way. The original article has been updated.

